# Workplace Violence in the Emergency Department: Case Study on Staff and Law Enforcement Disagreement on Reportable Crimes

**DOI:** 10.3390/ijerph19116818

**Published:** 2022-06-02

**Authors:** Sarayna S. McGuire, Aidan F. Mullan, Casey M. Clements

**Affiliations:** 1 Department of Emergency Medicine, Mayo Clinic, Rochester, MN 55902, USA; clements.casey@mayo.edu; 2 Department of Quantitative Health Sciences, Mayo Clinic, Rochester, MN 55902, USA; mullan.aidan@mayo.edu

**Keywords:** workplace violence, violence in healthcare, law enforcement, reportable violence, staff safety, occupational health

## Abstract

Violence in the emergency department (ED) remains underreported. Patient factors are often cited as a source of confusion in determining the culpability of perpetrators and whether to proceed with incident reporting. This study’s objective was to determine how ED staff at one academic medical center perceive certain clinical scenarios and how this compares to local law enforcement officers (LEO). An anonymous survey with 4 scenarios was sent to multidisciplinary ED staff at our academic medical center, as well as local LEO and inquired whether respondents considered any of the scenarios to be reportable as a crime. Chi-square analysis was used for comparison. The study was deemed exempt by the Institutional Review Board. A total of 261 ED staff and 77 LEO completed the survey. Both groups were equally likely to believe that a reportable crime occurred in Scenario 1, where a patient with dementia punches a nurse (LEO: 26.0% vs. ED: 31.4%, *p* = 0.44), and in Scenario 2, where an intoxicated patient spits at a phlebotomist (LEO: 97.4% vs. ED: 95.0%, *p* = 0.56). However, the two groups differed in Scenario 3, in which a patient with delirium makes verbal threats to a doctor (LEO: 20.8% vs. ED: 42.9%, *p* < 0.001), and Scenario 4, in which a patient’s parent throws a chair at a medical student (LEO: 66.2% vs. ED: 81.2%, *p* = 0.009). As health systems seek to improve workplace safety, it is important to consider the barriers to reporting violent incidents, including staff’s understanding of what acts may constitute reportable violence, as well as LEO understanding of the unique ED environment and patient responsibilities.

## 1. Introduction

Workplace violence is defined by the National Institute for Occupational Safety and Health (NIOSH) as “the act or threat of violence, ranging from verbal abuse to physical assaults, directed towards persons at work or on duty” [1]. Violence in the emergency department (ED) is a common and serious threat to staff, yet it remains underreported with prior literature indicating that 30% or fewer nurses and physicians go on to report incidents of workplace violence they experience [2,3,4,5,6,7,8,9,10,11,12,13]. There is a need to ascertain the true extent of this issue in healthcare to promote public awareness and target future violence mitigation efforts. Barriers to reporting are numerous and include longstanding complacency in thinking that violence is “part of the job,” [2] perceived or actual lack of support by management, fear of retaliation, lack of physical injury, cumbersome incident reporting processes, lack of time during a clinical shift to file a report, and confusion regarding what constitutes reportable violence in the setting of certain patient factors (e.g., psychosis, substance abuse, delirium, and dementia) [5]. Contributing patient factors may also make legal culpability less clear to law enforcement officers (LEO) involved in certain incidents.

We sought to better define this ultimate reporting barrier regarding staff perception of what constitutes reportable violence in the ED, and we sought to compare staff perception with that of local LEO, given their eventual involvement in incidents of violence against ED staff and potential for influence in reporting behaviors. Our primary study objective was to determine how ED staff at our academic medical center perceive certain clinical scenarios; more specifically, whether they would report the incidents presented in the scenarios, and how this perception compares to local LEO. Our secondary study objective was to determine whether respondent demographics (e.g., gender, years of experience, or clinical position) contributed to different perceptions of the scenarios.

## 2. Materials and Methods

### 2.1. Study Design and Setting

This descriptive prospective survey study took place between April–May 2020 in a city classified as a ‘small urban’ area approximately 55 miles from a major metropolitan area in the Midwestern United States. The study included both the local city police department that responded to nearly 5800 crimes in 2019 and the city’s primary emergency department (large, academic, Level 1 trauma center) with an average annual patient volume of 78,000 (prior to COVID-19) and 24/7 available hospital security presence.

Within the medical center, the official method of reporting incidents of workplace violence is through security personnel immediately when a threat or action has been identified and later in an employee incident report (EIR) when injuries are incurred, although prior research has demonstrated that staff report incidents through other (non-official) means as well, including supervisors, ED charge nurses, law enforcement, and the Medical Information Data Analysis System (MIDAS) [5]. The medical center has a policy to report all incidents of violence and has a designated violence mitigation group that maintains reports made through security and the EIR and supports staff who wish to report incidents of workplace violence.

### 2.2. Selection of Participants

The target population consisted of all sworn-LEO of the local police department (authorized strength of 150 sworn-LEO), as well as all multidisciplinary ED staff. Emergency department staff included clinicians (attending and resident physicians, and advanced practice providers (APP)), nursing staff (registered nurses and patient care assistants (PCA)), unit secretaries, electrocardiogram (ECG) and radiology technicians, phlebotomists, registration/finance staff, and security officers. After review by the institutional review board (IRB), the survey (described below) was emailed to the police department’s designated point of contact, who distributed it to LEO. The survey was also emailed to all distribution lists for the abovementioned ED target population by department and job type to anyone who might work in the ED, even occasionally. In total, the survey was sent electronically to approximately 960 hospital staff members. For both cohorts, the survey included a cover letter describing the study purpose, directions for participation, and information regarding informed consent. The questionnaire included a statement of informed consent at the beginning, and completion indicated participant consent for inclusion in the study. Two reminder notices were sent two weeks apart using the same method. The Mayo Clinic IRB reviewed this study (IRB 19-011681) and materials and deemed it exempt from the approval requirement.

### 2.3. Measurements

We developed an anonymous REDCap survey (Research Electronic Data Capture, Vanderbilt University, Nashville, TN, USA) [14] with four brief hypothetical patient encounter/case scenarios (Table 1). Respondents were asked to read each scenario and indicate whether they believed it constituted a reportable crime. All four scenarios demonstrated incidents of verbal abuse or physical assault against healthcare workers and included patient factors of dementia, substance abuse, delirium, and frustration with the care provided. Scenarios were developed by S.M. and C.C. based on commonly experienced violent encounters in the ED, as previously demonstrated by McGuire et al. [3], and were meant to introduce ambiguity to respondents with certain patient factors listed above. Standard demographic measures were collected, including gender, profession, primary shift worked (ED staff), and years of experience.

### 2.4. Outcomes

The primary outcome was the percentage of respondents in each cohort (LEO and ED staff), indicating whether each scenario constituted a reportable crime. The secondary outcome was the comparison of perceptions between both cohorts.

### 2.5. Data Analysis

Survey responses were summarized with frequency counts and percentages. We performed group comparisons of the survey responses using chi-squared tests and Fisher’s exact tests. *p*-values less than 0.05 were considered significant. We performed analyses using R version 3.6.2 (The R Foundation for Statistical Computing, Vienna, Austria).

## 3. Results

### 3.1. Characteristics of Study Subjects

Two hundred sixty-six survey responses were received from the ED staff cohort. Three respondents indicated management roles with no clinical duties, and two respondents did not complete the entirety of the survey; these five responses were excluded. The remaining 261 responses were included in the final analysis (estimated response rate of 27.2%). Seventy-seven survey responses were received from the LEO cohort and included in the final analysis; based on annual authorized strength, this corresponds to a 51.3% response rate. The demographics of both cohorts are provided in Table 2.

### 3.2. Perceptions of a Reportable Crime

As represented in Table 3, both groups were equally likely to believe that a reportable crime occurred in Scenarios 1 (LEO: 26.0% vs. ED: 31.4%, *p* = 0.44) and 2 (LEO: 97.4% vs. ED: 95.0%, *p* = 0.56).

The two groups differed in Scenarios 3 and 4. In Scenario 3, only 20.8% of LEO believed it represented a reportable crime, compared to 42.9% of ED staff (*p* < 0.001). Similarly, more ED staff believed that a reportable crime occurred in Scenario 4 compared to LEO (LEO: 66.2%, ED: 81.2%, *p* = 0.009).

There were no statistically significant differences in responses by gender in either the ED or LEO groups individually. Among both groups, those with fewer years of experience (0–4 years—51.6%) were more likely to believe Scenario 3 represented a reportable crime than those with more years of experience (5–10 years—34.7%; 11–20 years—30.0%; 21+ years—34.2%; *p* = 0.013; Table 4). Among LEO staff only, those with fewer years of experience (0–4 years) were more likely to believe Scenarios 1 (57.1%; *p* = 0.027) and 3 (42.9%; *p* = 0.037) represented a reportable crime compared to those with more years of experience. In Scenario 1, perceptions of violence were lower among clinicians and nurses (24/140, 16.0%) compared to all other positions (58/111, 52.3%; *p* < 0.001; Table 5). In Scenario 3, perceptions of violence were lower among clinicians and nurses (43/150, 28.7%) compared to all other positions (69/111, 62.2%; *p* < 0.001).

## 4. Discussion

The NIOSH defines workplace violence as “the act or threat of violence, ranging from verbal abuse to physical assaults, directed toward persons at work or on duty” [1]. This broad definition can lead to uncertainty for a unique subset of victims in healthcare who rely on clear definitions and diagnostic criteria in their line of work. Of note, this definition does not specifically account for underlying patient factors that may be present during agitated/violent patient encounters in the healthcare environment. Given this lack of actionable direction, there was disagreement between ED staff and LEO on what patient actions constitute a reportable crime in 2 of the 4 scenarios and variability in response among both groups.

Ensuring optimal reporting of violent incidents experienced in healthcare is essential—it allows institutions to maintain a comprehensive dataset that can be used to help design preemptive violence mitigation strategies and lead to reactionary policies and interventions (e.g., violent patient flags within the electronic medical record, change in staffing models, specialized staff training, etc.) while also providing targeted resources and support to victims [2].

This study helps confirm the longstanding belief that healthcare staff are uncertain what constitutes violence and/or criminal activity, particularly in unique situations where some may feel assailants are not responsible for their actions due to not being in full control of their faculties. In addition to the assailants’ mental state, there are also likely real or perceived barriers within law enforcement and the legal system to criminal culpability, such as likelihood to charge and prosecute in an already overwhelmed legal system, preconceived notions about healthcare violence within law enforcement or the legal system, likelihood to successfully prosecute biased by past experience of medical staff, and impressions about the potential severity or effectiveness of criminal punishments against the assailant. Indeed, the victim of an assault may be considering these downstream factors in determining whether or not to report the incident, even though this is generally not considered the responsibility of a victim in other non-healthcare settings. Such considerations can lead to uncertainty on the part of medical staff contributing to poor incident reporting, with prior data indicating less than one-third of nurses and physicians report incidents of workplace violence [2,3]. The authors suspect that patient factors such as dementia and delirium contributed to minimal ED staff indicating a reportable crime in Scenarios 1 and 3, respectively; however, future research should seek to clarify the underlying reason(s) for these responses. In particular, the few clinicians and nursing staff indicating that a crime occurred in Scenarios 1 (physical assault by punching) and 2 (physical assault by bodily fluids) should be emphasized, given prior research that has demonstrated these two disciplines are high-risk for physical assault [15]. Increased exposure to workplace violence may lead to the subsequent desensitization of violence perception within the workplace environment, similar to prior literature that has shown a desensitization to violence portrayed in the media and entertainment industry [16,17]. Furthermore, this study shows that our local LEO are equally uncertain in these non-straightforward clinical situations that may involve underlying delirium, dementia, substance abuse, or agitation. Delving into the factors that affect the likelihood of reporting an incident or the LEO reaction to an incident will require further study.

As healthcare workers look toward law enforcement to provide clarity or encouragement toward reporting of violent incidents experienced, the uncertainty that law enforcement has in these situations may directly influence reporting behaviors in healthcare staff. Additional work is needed to address whether LEO uncertainty in these situations has contributed to ED staff decisions not to report violent incidents experienced. Future efforts should also seek to clarify why LEO respondents do not believe reportable crimes are occurring in scenarios involving physical assault and verbal threats directed toward healthcare personnel. Perhaps this is due to LEO considering the full course of the criminal justice system and recognizing that scenarios involving underlying patient characteristics of dementia or delirium would result in more lenient sentences for perpetrators, or patients ultimately being found not guilty (or not competent to stand trial). In this study, however, the question posed to survey respondents was not whether the subject would ultimately be held responsible for their actions in the criminal justice system, but whether the scenario constituted a situation worth reporting on the victim’s behalf.

While the scenarios were hypothetical, they are not unrealistic in healthcare. Improvement interventions could be targeted at ED staff around the law and for LEOs to understand the unique environment of the ED and patient responsibilities. As health systems seek to improve workplace safety, it is important to consider the barriers to reporting violent incidents, including staff understanding of what acts may constitute reportable violence.

This study has several important limitations. To preserve the anonymity of ED staff, the study was sent to email distribution lists (DLs) and included some DLs with employees working in shared services that work in departments other than the ED (e.g., phlebotomy, and ECG and radiology technicians). Thus, it is not possible to know the actual number of employees from different disciplines who work in the ED, and we can only estimate an ED staff response rate for this study. We acknowledge this estimated response rate is low and thus an additional study limitation. That said, it does appear to be representative of the diversity in emergency department staff, particularly within patient care roles. While the response rate can be considered low, the breakdown of disciplines displayed in Table 2 demonstrates broad representation, and some disciplines that have relatively low numbers within the survey, such as advanced practice providers (*n* = 5), represent a significant portion of their available group (50% of 10 total possible). Nonpatient care staff may have been less likely to participate in the survey, as demonstrated in the registration/finance group where the number is quite low (*n* = 4), despite the staff size being a larger available potential total. Given the unique study objective, a pre-existing validated survey was not available for use in this study, and the survey was sent out at the advent of the COVID-19 pandemic, limiting attempts at pre-distribution survey validation. This study did not define to survey respondents what was meant by “reportable crime.” The interpretation of this term is highly subjective to individual respondents and likely differs between LEO and ED staff. In general, it was intended to convey the fact that the act may warrant either additional law enforcement or hospital security investigations. In future studies, this could be better defined to participants. The study did not ask respondents about previous acts of violence experienced and reported, but not follow-edup due to patient factors. As such, we cannot tell whether this experience influenced responses. This study was performed at one academic institution and police department in a Midwestern town and may not be generalizable to other institutions or geographic regions. However, the findings of uncertainty regarding what patient actions constitute a violent incident necessitating reporting are not dissimilar to other published studies.

## 5. Conclusions

Within our academic medical center, there was disagreement between ED staff and LEO on what patient actions constitute a reportable crime in 2 of the 4 scenarios and variability in response among both groups. As health systems seek to improve workplace safety, it is important to consider the barriers to reporting violent incidents, including staff understanding of what acts may constitute reportable violence, as well as LEO understanding of the unique environment of the ED and patient-care responsibilities. This represents an untapped opportunity for improved cooperation, communication, and training between healthcare systems and law enforcement institutions.

## Figures and Tables

**Table 1:** Case scenarios.

**Scenario 1:** An 85-year-old man with known dementia is transferred to the ED from his nursing home for back pain and is not oriented toward the year or his present location. He becomes agitated and punches a nurse attempting to obtain his vitals.
**Scenario 2:** A 25-year old man is brought into the ED by EMS with the complaint of broken teeth after getting into an altercation, and he appears to be intoxicated. He spits blood-tinged saliva into the face of the phlebotomist performing venipuncture.
**Scenario 3:** A 70-year-old female comes into the ED for abdominal pain. After a lengthy workup and prolonged stay, she begins showing signs of delirium and makes threats to find her doctor’s house upon discharge and harm him.
**Scenario 4:** A 1-year old is brought into the ED by his parents for a fever, rash, and upper respiratory symptoms. Upon hearing that a viral infection is the likely culprit and no further diagnostic studies will be ordered or antibiotics prescribed, the mother becomes increasingly angry and eventually throws a chair in the room, narrowly missing the medical student.

**Table 2:** Respondent Demographics.

	ED Cohort (*n* = 261) *n* (%)	LEO Cohort (*n* = 77) *n* (%)
**Gender**		
Male	104 (39.8%)	62 (80.5%)
Female	154 (59.0%)	8 (10.4%)
Prefer not to answer	3 (1.1%)	7 (9.1%)
**Primary Role**		
ED Staff		
Clinician	51 (19.5%)	
Attending physician	28 (10.7%)	
Resident physician	18 (6.9%)	
Advanced practice provider	5 (1.9%)	
Nursing Staff	99 (37.9%)	
Registered nurse	88 (33.7%)	
Patient care assistant	11 (4.2%)	
Phlebotomist	29 (11.1%)	
ECG/Radiology technician	24 (9.2%)	
Unit Secretary	12 (4.6%)	
Registration/Finance	4 (1.5%)	
Security	42 (16.1%)	
Management	0 (0%)	
LEO		77 (100%)
**Primary Shift**		
Day	68 (26.1%)	
Evening	45 (17.2%)	
Night	53 (20.3%)	Not asked
Rotating	95 (36.4%)	
**Years of Experience**		
0–4 Years	86 (33.0%)	7 (9.1%)
5–10 Years	57 (21.8%)	15 (19.5%)
11–20 Years	74 (28.4%)	26 (33.8%)
21+ Years	44 (16.9%)	29 (37.7%)

**Table 3:** Number (percentage) of respondents indicating the scenario demonstrated a reportable crime.

	ED Staff (*n* = 261) *n* (%)	LEO (*n* = 77) *n* (%)	*p*-Value
**Scenario 1**	82 (31.4%)	20 (26.0%)	0.439
**Scenario 2**	248 (95.0%)	75 (97.4)%	0.564
**Scenario 3**	112 (42.9%)	16 (20.8%)	<0.001
**Scenario 4**	212 (81.2%)	51 (66.2%)	0.009

**Table 4:** Number (percentage) of respondents indicating the scenario demonstrated a reportable crime by years of experience.

All Participants					
	**0–4 Years** **(** ***n*** **= 93)**	**5–10 Years** **(** ***n*** **= 72)**	**11–20 Years** **(** ***n*** **= 100)**	**21+ Years** **(** ***n*** **= 73)**	***p-*** **Value**
**Scenario 1**	35 (37.6%)	23 (31.9%)	21 (21.0%)	23 (31.5%)	0.085
**Scenario 2**	89 (95.7%)	67 (93.1%)	97 (97.0%)	70 (95.9%)	0.665
**Scenario 3**	48 (51.6%)	25 (34.7%)	30 (30.0%)	25 (34.2%)	0.013
**Scenario 4**	72 (77.4%)	56 (77.8%)	76 (76.0%)	59 (80.8%)	0.901
**ED Staff Only**					
	**0–4 Years** **(** ***n*** **= 86)**	**5–10 Years** **(** ***n*** **= 57)**	**11–20 Years** **(** ***n*** **= 74)**	**21+ Years** **(** ***n*** **= 44)**	***p-*** **Value**
**Scenario 1**	31 (36.0%)	19 (33.3%)	19 (25.7%)	13 (29.5%)	0.541
**Scenario 2**	82 (95.3%)	53 (93.0%)	71 (95.9%)	42 (95.5%)	0.880
**Scenario 3**	45 (52.3%)	22 (38.6%)	29 (39.2%)	16 (36.4%)	0.192
**Scenario 4**	67 (77.9%)	48 (84.2%)	58 (78.4%)	39 (88.6%)	0.402
**Law Enforcement Officers Only**					
	**0–4 Years** **(** ***n*** **= 7)**	**5–10 Years** **(** ***n*** **= 15)**	**11–20 Years** **(** ***n*** **= 26)**	**21+ Years** **(** ***n*** **= 29)**	***p-*** **Value**
**Scenario 1**	4 (57.1%)	4 (26.7%)	2 (7.7%)	10 (34.5%)	0.027
**Scenario 2**	7 (100%)	14 (93.3%)	26 (100%)	28 (96.6%)	0.584
**Scenario 3**	3 (42.9%)	3 (20.0%)	1 (3.8%)	9 (31.0%)	0.037
**Scenario 4**	5 (71.4%)	8 (53.3%)	18 (69.2%)	20 (69.0%)	0.705

**Table 5:** Number (percentage) of respondents indicating the scenario demonstrated a reportable crime by ED staff position.

	Scenario 1	Scenario 2	Scenario 3	Scenario 4
Clinician	8 (15.7%)	47 (92.2%)	19 (37.3%)	39 (76.5%)
Nursing Staff	16 (16.2%)	95 (96.0%)	24 (24.2%)	84 (84.8%)
Phlebotomist	12 (41.4%)	28 (96.6%)	18 (62.1%)	28 (96.6%)
ECG/Radiology Technician	8 (33.3%)	22 (91.7%)	12 (50.0%)	19 (79.2%)
Unit Secretary	8 (66.7%)	12 (100.0%)	7 (58.3%)	9 (75.0%)
Registration/Finance	2 (50.0%)	4 (100.0%)	4 (100.0%)	3 (75.0%)
Security	28 (66.7%)	40 (95.2%)	28 (66.7%)	30 (71.4%)
*p*-Value	<0.001	0.854	<0.001	0.163

## Meeting

An abstract with limited data from this study was presented at the ACEP 2020 virtual meeting.

## Abbreviations

ECG	Electrocardiogram
ED	Emergency Department
LEO	Law Enforcement Officer
PCA	Patient Care Assistant(s)
